# Thioguanine Induces Apoptosis in Triple-Negative Breast Cancer by Regulating PI3K–AKT Pathway

**DOI:** 10.3389/fonc.2020.524922

**Published:** 2020-10-30

**Authors:** Daoyu Zhang, Xinglan An, Qi Li, Xiaxia Man, Meiran Chu, Hao Li, Nan Zhang, Xiangpeng Dai, Hao Yu, Ziyi Li

**Affiliations:** ^1^ Key Laboratory of Organ Regeneration and Transplantation of Ministry of Education, First Hospital, Jilin University, Changchun, China; ^2^ College of Animal Science, Jilin University, Changchun, China

**Keywords:** ****triple-negative breast cancer, transcriptome, thioguanine, methylation, PI3K–AKT

## Abstract

Triple-negative breast cancer (TNBC) is notoriously difficult to treat due to the lack of biological targets and poor sensitivity to conventional therapies. Chemotherapy is the main clinical therapy, but the effective screening strategy for chemotherapy drugs is poorly investigated. Drug repositioning has been the center of attention in recent years attracting numerous studies. Here, we firstly found multiple common features between leukemia and TNBC by analyzing the global transcriptome profiles based on the transformed comparison data from NCI60. Therefore, we investigated the role of the classic leukemia drug thioguanine (6-TG) in TNBC cancer cells. Our results indicated that 6-TG inhibited cell proliferation and tumor cell progression by suppressing PI3K–AKT pathway *via* downregulating the DNA methylation level of *PTEN*. Moreover, apoptosis was induced *via* the activation of PI3K-AKT downstream *TSC1* and the downregulation of methylation levels of *DAXX*, *TNF*, *FADD* and *CASP8* *etc.* These findings indicated 6*-*TG exerts its anti-tumor effects *in vitro* and *in vivo* through regulating the DNA methylation levels of genes involved in PI3K–AKT and apoptosis pathway. Meanwhile, our study suggested that transcriptome-based drug screening has potential implications for breast cancer therapy and drug selection.

## Introduction

TNBC is a breast cancer subtype that does not clinically express significant levels of the estrogen receptor (ER), progesterone receptor (PR), and human epidermal growth factor receptor 2 (HER2), representing only 15–20% of breast cancer cases (1–3). However, TNBC is the most aggressive breast cancer type without any approved targeted therapy (4, 5). Currently, chemotherapies are the only therapeutic treatment for TNBC. Therefore, drug repurposing and repositioning have attracted increasing attention in recent years (6, 7), such as thalidomide, a drug for morning sickness but currently is used for the treatment of multiple myeloma (8). Therefore, exploration of effective drug selection strategy for TNBC has become increasingly important. Recent studies have attempted to find the tumor pathogenesis through high throughput sequencing, but the integration of transcriptome maps of various tumors for drug discovery remains slow.

Recently, it is widely accepted that human oncovirus can induce malignant tumors after a long latency and in conjunction with environmental factors. Over 30 years ago, human T-cell leukemia virus type 1 (HTLV-1) was identified as the first human cancer-causing retrovirus (9). Since then, a variety of tumor-associated viruses have been discovered, including Epstein–Barr virus (10), classified as Class I carcinogen by the International Agency for Research on Cancer (IARC) (11, 12), human papillomavirus (HPV), mouse breast cancer virus-like virus (MMTV-like). These viruses are involved in several different lymphoid and epithelial malignancies, including Burkitt’s lymphoma (BL), Hodgkin’s disease, non-Hodgkin’s lymphoma, nasopharyngeal, gastric cancer, and breast cancer. Recent publications have shown that EBV, human papillomavirus (HPV), mouse breast oncovirus-like virus (MMTV-like), and polyamorous JCV can serve as cofactors in the oncogenic process in breast cancer and increase the aggressiveness of the disease (13). Therefore, we predicted that the occurrence of TNBC was probably related to oncovirus infection, based on its pathogenetic similarity to that of leukemia/lymphoma.

High-throughput sequencing technology has been considered as a promising and effective tool in discovering meaningful genetic and epigenetic variations during cancer development and identifying biomarkers for cancer diagnosis or prognosis (14). To date, less efforts have attempted to explore pathogenic mechanisms by comparing the transcriptional characteristics between breast cancer and other cancers. Here, we analyzed transcriptome profiles between breast cancer and leukemia/lymphoma and identified similar expression profiles in some pathways including PI3K–AKT pathway and viral infection pathways, which might imply the potential application of leukemia/lymphoma chemotherapeutics in TNBC treatment. Therefore, this study was aimed to evaluate the anti-tumor effect of the classic leukemia drug 6-TG on TNBC *in vitro* and *in vivo* and to elucidate the underlying mechanism(s). Our results will provide novel therapeutic options to TNBC patients.

## Material and Methods

### Cells and Chemicals

The human TNBC cell line MDA-MB-231 was obtained from Northeast Forestry University; MDA-MB-231-Luciferase was purchased from ORIGENE (Beijing, China); normal breast epithelial cell line MCF-10A and human TNBC cell line HCC1937 were purchased from Procell (Wuhan, China). TNBC cells were cultured in RPMI 1640 medium (Gibco, Grand Island, USA) supplemented with 10% fetal calf serum, 100 IU/ml of penicillin and 100 μg/ml of streptomycin (HyClone, Los Angeles, USA) and MCF-10A Cell Culture Medium (Procell, Wuhan, China). The cells were cultured in a humid environment with 5% CO2 at 37°C. All human cell lines have been authenticated using STR profiling within the last three years. All experiments were performed with mycoplasma-free cells.

Thioguanine (6-TG), CX-6258 HCl, MI-463, and Docetaxel were purchased from Selleck Chemicals (Selleck, Houston, USA, 154-42-7). Dimethyl sulfoxide (DMSO), polyethylene glycol (PEG300), Tween80, and ddH2O were purchased from Sigma-Aldrich Co.

### Proliferation Assay

Cells were treated with different concentrations of 6-TG (or CX-6258 HCl, or MI-463) then were subjected to the Cell Counting Kit-8 assay according to the manufacturer’s protocol (DOjinDO, JAPAN). Briefly, 6.5 × 10^3^ cells were seeded per well in a 96-well plate and treated with 6-TG at indicated concentration. Thereafter, CCK-8 solution was added, and the Absorbance (A) was measured using a microplate reader (BIO-RAD, California, USA) at 450 nm. IC50 values were calculated using GraphPad Prism 6.01 software. IC20 values were calculated using SPSS Statistics.

### Colony Formation Assay

Four hundred cells per well were randomly plated in 6-well plates and were cultured in vehicle (control) or 6-TG (or CX-6258 HCl, or MI-463) containing medium. Triplicate wells were used for each group. The cells were cultured for 8–9 days until the cells grew up to 50 cells, which was considered as one colony. Then cells were stained with Giemsa and counting (SIGMA, Illinois, USA).

### Migration Assay

Cells were seeded in 12-well plates and grew to confluence, followed by wound creation. Then cells were incubated with 6-TG or vehicle. Cell migration images were collected every 6 h until 24 h. Triplicate wells were used for each group. The migration rate was calculated using ImageJ software.

### Apoptosis Assay

Cells were stained using the TransDetectTM Annexin V-EGFP/PI Cell Apoptosis Detection Kit (TRAN, Beijing, China). Briefly, 2 × 10^5^ cells were seeded in each well and treated with 2.5 μM 6-TG (or 3.9 μM CX-6258 HCl, or 13.9 μM MI-463) or vehicle. The cells were collected, followed by the addition of Annexin-V-FITC or propidium iodide (PI) or both of them to the cell suspension, then incubated for 15 min. The stained samples were analyzed by flow cytometry using a FACSCalibur flow cytometer (BD FACSCanto II, New Jersey, USA), and FlowJo VX was used to analyze the data.

### Immunofluorescence Assay

Cells treated with 6-TG or vehicle were fixed and permeabilized with 0.1% Triton X-100 in PBS and were blocked with 1% bovine serum albumin, followed by incubation with the DNMT1 antibody (Genetex, Southern California, USA) overnight. Next, the cells were stained with secondary antibody (Proteintech, Chicago, USA), followed by staining with 4,6-diamidino-2-phenylindole to visualize the nuclei. The analysis of fluorescence intensity was analyzed using ImageJ software (National Institutes of Health, Bethesda, MD) based on procedures described elsewhere (15, 16).

### RT2 Profiler PCR Array

RNA was isolated from about 2 × 10^7^ cells using the ALLPrep DNA/RNA Mini Kit (QIAGEN, Germany), and cDNA was synthesized using the Transcript one-step gDNA Removal and cDNA Synthesis SuperMix. qPCR was performed using SYBR premix Ex Taq (Takara, Japan) on a LightCyclerR 96 Real-Time PCR System (17). The Human PI3K–AKT RT2 and Human Apoptosis RT2 profiler PCR arrays were performed according to the manufacturer’s instructions. Primers sequences are listed in **Table S1** and **Table S2**.

### RNA Preparation and RNA-Seq

Total RNA was extracted with TRIzol (Invitrogen, California, USA) reagent following the manufacturer’s instructions. The insert size was assessed using the Agilent Bioanalyzer 2100 system, and qualified insert sizes were accurately quantified using the StepOnePlus™ Real-Time PCR System (Library valid concentration >10 nM). Sequencing libraries were generated using the NEBNext^®^ Ultra™ RNA Library Prep Kit for Illumina^®^ (USA, NEB) following the manufacturer’s recommendations, and index codes were added to attribute sequences to each sample (GEO number: GSE137418).

### Western Blotting

Cells were lysed in RIPA buffer after treatment with 2.5 μM 6-TG or vehicle. Equal amounts of protein were resolved by SDS-PAGE, then analyzed by western blotting with the indicated antibodies: anti-GAPDH (Proteintech, 60004-1-Ig), anti-DNMT1 (Genetex, GTX116011), anti-pAKT (abcam, ab38449), anti-AKT (abcam, ab8805) and HRP-conjugated anti-rabbit and anti-mouse secondary antibodies (Proteintech, SA00006-4). The Human Apoptosis Array Kit (R&D, California, USA) was used for apoptosis array. The complete information about the antibodies is shown in **Table S3**.

### DNA Methylation Detection

DNA was isolated from 10^7^ cells using the DNeasy Blood and Tissue Kit (QIAGEN, Germany). Approximately, 500 ng of genomic DNA from each sample was used for sodium bisulfite conversion using the EZ DNA methylation Gold Kit (Zymo Research, USA) following the manufacturer’s standard protocol. Genome-wide DNA methylation was assessed using the Illumina Infinium HumanMethylation850K BeadChip (Illumina Inc., USA) according to the manufacturer’s instructions. The array data (IDAT files) were analyzed using the ChAMP package in R to derive the methylation level.

### Tumor Cell Inoculation and Injections

NOD-SCID mice (3–4 weeks) were purchased from Beijing Vital River Laboratory Animal Technology Co., Ltd. Next, 1 × 10^6^ exponentially growing MDA-MB-231 cells with luciferase-labeled (MDA-MB-231-Luc) were injected subcutaneously into the left groin of each NOD-SCID mouse, five mice each group. Tumor-bearing mice were injected with 1.5 mg/kg of 6-TG or solvent five times a week, 20 mg/kg Docetaxel once a week after tumors were inoculated for 7 days. When the tumors became palpable, a digital caliper was used to measure the tumor size every two days. Additionally, the tumor fluorescence intensity was detected with IVIS^®^ Lumina III (PerkinElmer, USA) weekly. Tumor volumes (Vs) were calculated as ellipsoids (V = π/6 · l ·w ^2^, where l is the length, w is the width). Experiments ended when the tumor volumes reached approximately 1.5 cm^3^ or earlier if necrotic. The mice were killed by CO_2_ asphyxiation. All mice were raised in a specific pathogen-free environment with free access to food and water and received care in accordance with the guidelines outlined in the Guide for the Care and Use of Laboratory Animals. All procedures were approved by the Jilin University Animal Care and Use Committee.

### HE Staining and Immunohistochemistry Staining

Allograft tumors and the livers and kidneys were dissected and fixed in 10% (v/v) neutral-buffer formalin. They were then dehydrated in ascending grades of ethanol and xylene and embedded in paraffin wax. Thereafter, the sections (4 μm) were cut with a microtome (Leica, Germany). Anti-DNMT1 antibody, anti-caspase-3 antibody and goat anti-rabbit antibody IgG (BOSTER, Wuhan, China) were used for immunostaining. For HE staining, after deparaffinization and rehydration, 4-μm sections were stained with hematoxylin solution for 5 min followed by 5 dips in 1% acid ethanol and then rinsing in water. Next, the sections were stained with eosin solution for 3 min, followed by dehydration with graded alcohol and clearing in xylene.

### Statistical Analysis

Different statistical tools were used to analyze the data. The experiments were repeated at least two times in triplicate. In the graphs, the results are presented as mean ± SEM. Statistical analysis was performed using GraphPad Prism 6.01 (GraphPad Software, USA). Student’s t-test was used to compare the control and experimental groups. For all comparisons, *p < 0.05, **p < 0.01, and ***p < 0.001 were considered to show a significant difference.

### Data Availability

The data of RNA-seq was uploaded on GEO, the number is GSE137418. Other data is available from the corresponding author upon reasonable request.

## Results

### Analysis of Pathogenic Molecular Pathways Between Breast Cancer and Leukemia

**Figure 1:**
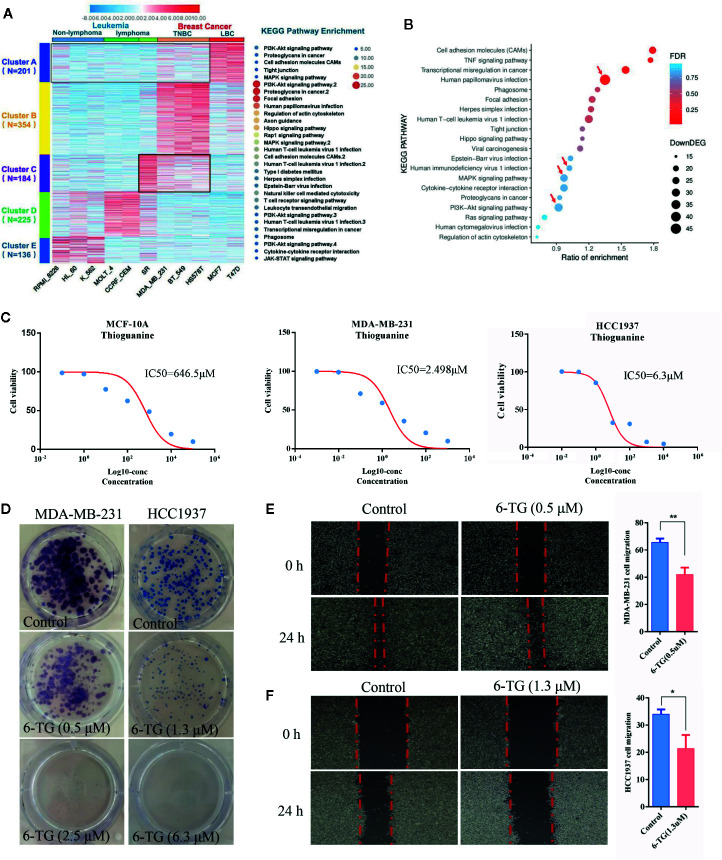
Analysis of the pathogenic molecular pathways between breast cancer and leukemia. **(A)** Clustering was performed using IDEP 8.0. Six lymphoma cell lines (non-lymphoma: RPMI-8226, HL-60, K-562; lymphoma: MOLT-4, CCRF-CEM, SR) and 5 breast cancer cell lines (TNBC subtype: MDA-MB-231, BT549, HS578T; luminal subtype: MCF7, T-47D) were selected from the dataset GSE32474: NCI-60 cancer cell line panel. The colors in the map displayed the relative normalized values of the top 1,000 differentially expressed genes (DEGs) within six leukemia cell lines and five breast cancer cell lines; blue indicates the lowest expression, and red indicates the highest expression. The color scale bar was shown at the top of the heatmap. The number of genes enriched in the KEGG pathway in each cluster was listed on the right side of the heatmap, and the number of genes in each cluster was displayed on the left side of the heatmap. **(B)** KEGG pathway enrichment of up-DEGs between MDA-MB-231 and MCF-10A. **(C)** IC50 values of 6-TG in MDA-MB-231, HCC1937 and MCF-10A cells. X-axis presented the Log10-concentration, and the y-axis presented the cell survival rate. **(D)** Colony formation of MDA-MB-231 and HCC1937 cells on the IC20 concentration (0.5 μM in MDA-MB-231 or 1.3 μM in HCC1937) and IC50 concentration (2.5 μM in MDA-MB-231 or 6.3 μM in HCC1937). **(E)** Cell migration of MDA-MB-231 cells on the IC20 concentration. **(F)** Cell migration of HCC1937 cells on the IC20 concentration. (All values from three independent experiments are quantified as mean ± SD, **p < 0.01, *p < 0.05).

To explore the potential correlation between breast cancer and leukemia, we analyzed the global transcriptome profiles based on the transformed comparison data from NCI60. As shown in **Figure 1A**, the genes in Cluster C are shared by TNBC and lymphoma (SR: large-cell immunoblastic lymphoma). These 184 genes are mainly involved in cell adhesion molecules, human T-cell leukemia virus 1 infection, herpes simplex infection, and Epstein–Barr virus infection. Cluster B is a TNBC-specific cluster containing 354 genes, which are mainly related to pathways such as focal adhesion, PI3K–AKT signaling, human papillomavirus infection, human T-cell leukemia virus 1 infection. Moreover, compared with MCF-10A, the enrichment of highly expressed genes in MDA-MB-231 cells was consistent with the results of Clusters B and C in the TNBC (**Figure 1B**). Human papillomavirus infection (n > 40), PI3K–AKT signaling pathway (n > 30) and human T-cell leukemia virus 1 infection (n > 20) had the highest degrees of gene enrichment. Based on the summarized data, we propose that some drugs targeting leukemia might have a similar role in inhibiting TNBC progression.

Next, the 6-TG, a well-studied classic medicine for the treatment of leukemia, was used as a candidate drug to confirm our proposal and to evaluate its role in TNBC cells. To establish a proper treatment dose, MDA-MB-231, HCC1937, and MCF-10A cells were treated with various concentrations of 6-TG. The cell viability assay revealed that the IC50 values of 6-TG in MDA-MB-231, HCC1937, and MCF-10A cells were 2.489, 6.3, and 646.5 μM on 48 h, respectively (**Figure 1C**). Additionally, the cytotoxic effects of 6-TG on MDA-MB-231 cells were shown in a dose-dependent manner (**Figure S1**). We observed that, in comparison with the control (vehicle), treatment of 6-TG on the IC20 concentration (0.5 μM in MDA-MB-231 or 1.3 μM in HCC1937) or IC50 concentration (2.5 μM in MDA-MB-231 or 6.3 μM in HCC1937) led to a significant inhibition of colony formation (**Figure 1D**) in MDA-MB-231 and HCC1937 cells. In addition, as shown in **Figures 1E, F**, cell migration was inhibited under treating with 6-TG on the IC20 concentration. Based on these data, 2.5 μM 6-TG was used in MDA-MB-231 in subsequent experiments. Altogether, these data showed that 6-TG was effective in inhibiting human MDA-MB-231 and HCC1937 cells growth.

### Signaling Pathways Altered by 6-TG Treatment in MDA-MB-231 Cells

**Figure 2:**
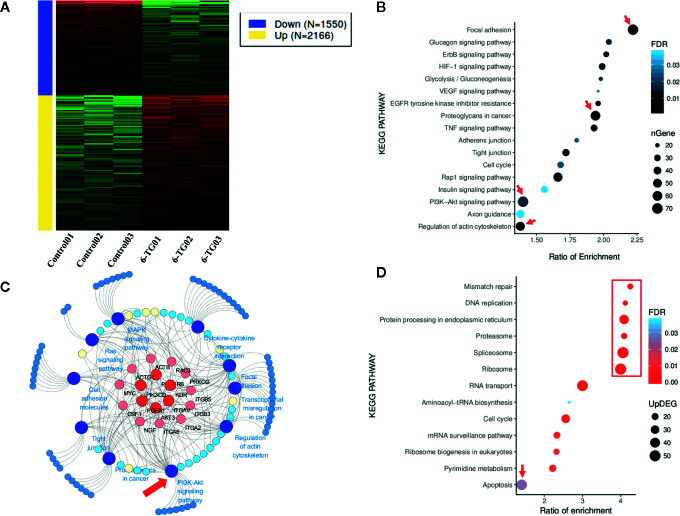
Genes in different signaling pathways altered by 6-TG treatment in MDA-MB-231 cells. **(A)** Heatmap of gene expression differences in MDA-MB-231 cells. The red color indicates upregulated genes, and the blue color indicates downregulated genes. **(B)** The bubble plot of pathway enrichment of downregulated genes. **(C)** The cancer development pathway gene network. The network was generated using 10 downregulated cancer pathways and their enriched DEGs. **(D)** The bubble plot of the pathway enrichment of upregulated genes. Network graphs were prepared by Cytoscape 3.6.1.

To explore the underlying mechanisms that 6-TG could inhibit TNBC cell growth, we carried out RNA-seq study using the samples treated with or without 2.5 μM 6-TG in MDA-MB-231. The heatmap showed obvious changes in gene expression profile between the two groups (**Figure 2A**) with 2,166 upregulated genes and 1,550 downregulated genes in response to 6-TG treatment. The downregulated genes were mainly involved in focal adhesion, proteoglycans in cancer, and PI3K–AKT signaling pathway (**Figure 2B**). We structured a network of these genes and found that the PI3K–AKT pathway contained the most genes shared with others (**Figure 2C**). Additionally, the virus infection pathways were almost completely inhibited by 6-TG (**Figure S2**). The pathways related to mismatch repair, DNA replication, and proteasome displayed higher gene ratios in the upregulated genes, while pathways involved in cell apoptosis showed the highest degree of gene enrichment (**Figure 2D**). These results suggested that 6-TG exerted anti-tumor activity by downregulating the genes in virus infection pathways, cancer development pathways and upregulating the genes in mismatch repair and apoptosis. It is worthy of attention that the PI3K–AKT pathway, which was abnormally highly activated in TNBC, was inhibited by 6-TG.

### PI3K-AKT Pathway Was Attenuated by 6-TG Treatment in MDA-MB-231 Cells

**Figure 3:**
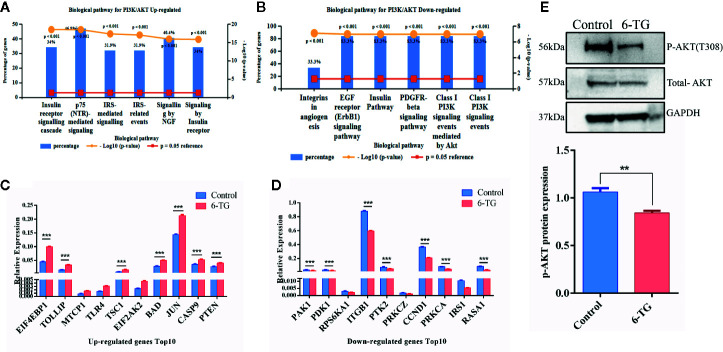
PI3K–AKT pathway was blocked by 6-TG in MDA-MB-231 cells. **(A)** The top five pathways of upregulated genes were enriched (ranked by P-value using the FunRich 3.0 software). **(B)** The top five pathways of downregulated genes were enriched (ranked by P-value using the FunRich 3.0 software). **(C)** The top 10 upregulated genes in the PI3K–AKT pathway. **(D)** The top 10 downregulated genes in the PI3K–AKT pathway. **(E)** Western blotting of p-AKT (T308) and total AKT. (All values from three independent experiments are quantified as mean ± SD, **p < 0.01, ***p < 0.001).

Based on the results of RNA-seq, the expression profile of members in the PI3K–AKT pathway was investigated by qPCR array. The expression of 53 genes was upregulated, and 31 genes were downregulated (**Figure S3**). The pathway enrichment of upregulated genes analyzed by FunRich showed that the genes were mainly involved in p75 mediated signaling (46.8%) (**Figure 3A**). However, the downregulated genes were mainly involved in PI3K signaling events (83.3%) and EGF receptor signaling pathway (83.3%) (**Figure 3B**). The top 10 upregulated DEGs were *PTEN, TSC1, BAD, JUN, CASP9, EIF2AK2, EIF4EBP1, TOLLIP* (**Figure 3C**), while the top 10 downregulated DEGs were *ITGB1, PTK2, CCND1, PAK1, PDK1, PRKLA, RASA1* (**Figure 3D**). We used Western blotting analysis to confirm that p-AKT (T308) was significantly decreased by 6-TG treatment (**Figure 3E**). These results suggested that 6-TG blocked the PI3K–AKT pathway by activating *PTEN*, and the activation of downstream genes such as *BAD, CASP9, TSC1* also supported these data. More importantly, the upregulation of tumor suppressor genes in the PI3K–AKT pathway such as *TSC1*, *CASP9*, and *PTEN* indicates a possible role of 6-TG in promoting apoptotic signaling pathways.

### Apoptosis Was Induced by 6-TG in MDA-MB-231 Cells

**Figure 4:**
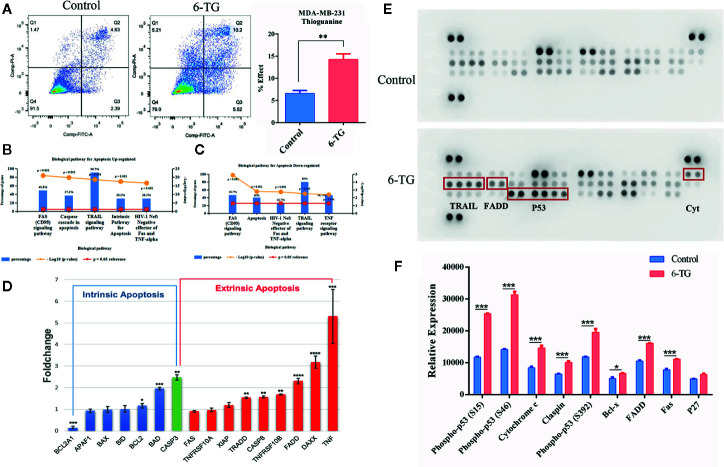
Apoptosis was induced by 6-TG in MDA-MB-231 cells. **(A)** Cell apoptosis was detected by flow cytometry. **(B)** The top five pathways that upregulated genes were enriched in. **(C)** The top five pathways that downregulated genes were enriched in. **(D)** Relative expression levels of apoptosis-related genes. **(E)** Apoptosis antibody array analysis. **(F)** The relative protein expression analysis is on the bottom. The scatter plot represented the relative expression of proteins. (All values from three independent experiments are presented as mean ± SD, *p < 0.05, ** p < 0.01, ***p < 0.001, ****p < 0.0001).

To further investigate whether 6-TG inhibits TNBC cell growth *via* triggering apoptosis signaling, we assessed apoptotic cell death by Annexin V/PI staining (**Figure 4A**). The ratios of apoptotic cells were 7.02% in the control group and 15.72% in the 6-TG groups, respectively, indicating 6-TG treatment led to a significant increase of apoptosis (P < 0.001). To explore the underlying molecular basis of effects of 6-TG on cell apoptosis, we carried out apoptosis PCR array, and discovered 67 upregulated genes and 17 downregulated genes (**Figure S4-A**). The upregulated genes were mainly involved in TRAIL signaling pathway (90.7%) and FAS signaling pathway (48.8%) (**Figure 4B**), while the majority of downregulated genes were related to TRAIL signaling pathway (80%), TNF signaling pathway (46.7%) (**Figure 4C**). Results in **Figure S4-B** and **Figure S4-C** displayed the top 20 upregulated or eight downregulated DEGs, respectively. Moreover, the genes in the extrinsic apoptosis pathways were significantly increased than those related to intrinsic apoptosis pathways (**Figure 4D**). Meanwhile, the results of the antibody array for apoptosis showed that proteins related to apoptosis were increased after 6-TG treatment (**Figure 4E**). The top 10 upregulated proteins with the highest fold-change were P53, Cytochrome c, FADD, Fas *etc.* (**Figure 4F**). These results indicated that 6-TG induced cell apoptosis, mainly through regulating TRAIL signaling.

### Decreased DNA Methylation in Apoptosis-Related Genes in MDA-MB-231 Cells

**Figure 5:**
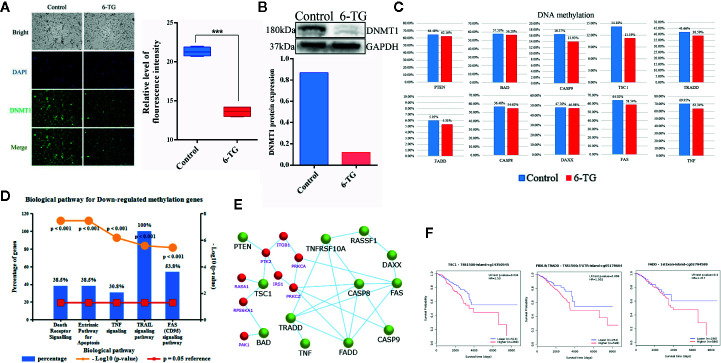
Decreased DNA methylation in apoptosis-related genes in MDA-MB-231 cells. **(A)** Immunofluorescence staining of DNMT1 in MDA-MB-231 cells. The nuclei (blue) were stained with DAPI. (***p < 0.001). **(B)** Western blotting analysis of DNMT1 protein (***p < 0.001). **(C)** The DNA methylation levels of genes involved in apoptosis and the PI3K–AKT pathway. **(D)** The top five pathways of downregulated DNA methylation genes were enriched. **(E)** Network of genes with downregulated methylation genes and genes (red) of the PI3K–AKT pathway. **(F)** Survival curves of the DNA methylation level of *TSC1, FADD*, and *TRADD* were analyzed by MethSurv. (All values from three independent experiments are quantified as mean ± SD, ***p < 0.001).

Given the causal relationship between DNMT1, DNA methylation, and 6-TG, we interrogated the role of 6-TG in regulating the DNA methylation level of apoptosis-related genes mentioned above. Immunofluorescence (IF) staining showed that the DNMT1 protein level in the 6-TG group (13.62 ± 0.3347) was lower than that in the control group (21.32 ± 0.2879) (**Figure 5A**), which is consistent with the result tested by western blotting analysis indicating a reduced protein level of DNMT1 upon 6-TG treatment (**Figure 5B**) (P < 0.001). Furthermore, the methylation differences were also measured by Illumina’s Methylation EPIC850K BeadChip. The volcano plot showed 2,242 and 6,084 genes with down- or upregulated methylation levels, respectively (FC > 2; P < 0.01) (**Figure S5**). The DNA methylation level of *PTEN* was decreased; meanwhile, the DNA methylation levels of *BAD, CASP9, TSC1, TNF, DAXX, FADD, TRADD, CASP8 and FAS* were all reduced by 6-TG treatment (**Figure 5C**). Moreover, the genes with downregulated DNA methylation were mainly enriched in TRAIL, FAS, and extrinsic signaling pathways (**Figure 5D**). These results proved 6-TG regulating the DNA methylation levels of apoptosis-related genes mentioned in **Figure 3C** and **Figure 4D**. To illustrate the link between apoptosis-related genes and genes involved in the PI3K–AKT pathway, we constructed a main effective network of these genes and discovered an interaction between them (**Figure 5E**). We further detected the survival curves of the CpG sites of these genes (**Figure 5F**). The plots showed that the survival of breast cancer patients with reduced methylation of *TSC1* (cg14350545), *FADD* (cg02794589), and *TRADD* (cg05178604) was significantly improved. These results altogether showed that the reduced methylation of *PTEN* which would active *PTEN* will inhibit PI3K–AKT pathway and activated *BAD, CASP9, TSC1 via* interaction between them (18). Moreover, the reduced DNA methylation levels of these apoptosis promoting genes will activate them and subsequently induce apoptosis.

### 6-TG Treatment Retarded Tumor Growth *In Vivo*

**Figure 6:**
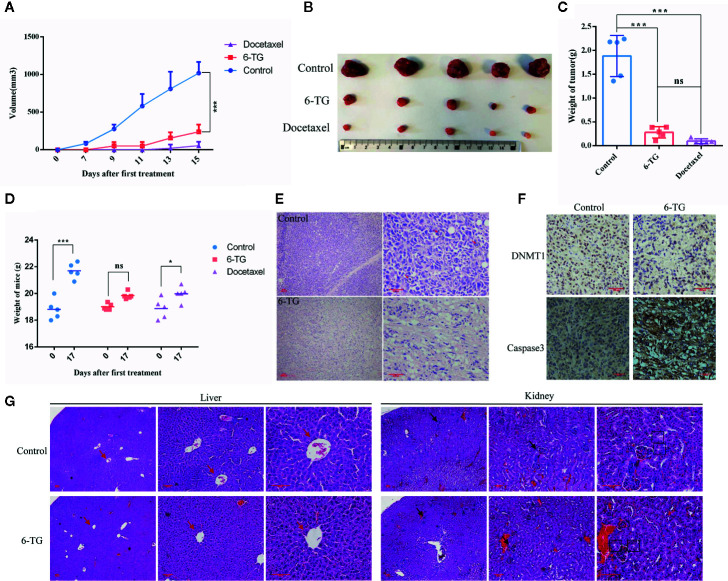
6-TG treatment retarded tumor growth *in vivo*. **(A)** Drug-response curves of the MDA-MB-231 tumors. The data points represent the mean values  ±  SD of five independent experiments. The blue, red, and violet lines represent the control group, 6-TG group and docetaxel group,respectively. **(B)** Tumor morphology of the control group (top), 6-TG group (middle) and docetaxel group (bottom). **(C)** Weights of the final tumors. **(D)** Weight of mice during the treatment. X-axis indicated the days after implantation; Y-axis indicated the average weights of every groups. **(E)** The tumor sections were stained with HE. The positions indicated by the red arrow are pathological mitotic events. (Scale bar: 200 μm) **(F)** Immunohistochemical staining of DNMT1 and caspase-3 antibodies in tumors. (Scale bar: 200 μm). **(G)** The livers and kidneys from tumor bearing mice in control group and 6-TG group were stained with HE (Scale bar: 400 μm). The red arrows pointed the central veins in livers. The black arrows pointed the renal corpuscles, and the black boxes pointed the proximal convoluted tubules. (All values from three independent experiments are quantified as mean ± SD, *p < 0.05, ***p < 0.001, ns indicates no significant).

To further investigating the negative regulation of 6-TG on tumor cell growth, we performed the xenograft experiment *in vivo*. Docetaxel was used as a positive control. As shown in **Figures 6A, B**, 6-TG treatment significantly retarded the growth of tumor xenografts in mouse models, and its effect was similar to that of docetaxel (***p < 0.001). Meanwhile, the fluorescence signal intensities were weaker, and the fluorescence area sizes were smaller in the 6-TG group than that in the control group from day 20 after inoculation to the last detection (**Figure S6**). Consistently, the tumor weights were significantly reduced after 6-TG treatment (***p < 0.001) (**Figure 6C**, and 6-TG had no significant effect on body weight of the animals during the treatment (**Figure 6D**). Hematoxylin–eosin (HE) staining showed several pathological mitotic events in the control group but not in the 6-TG group (**Figure 6E**), which further confirmed that tumor growth was inhibited. To further investigate the underlying mechanisms, immunohistochemistry staining was performed; the DNMT1 expression reduced, and caspase-3 expression increased in 6-TG treated tumors (**Figure 6F**). Furthermore, the HE staining of livers and kidneys of tumor bearing mice showed no significant difference between control group and 6-TG group. Specifically, the structures of the central vein (red arrows), hepatic plate, and hepatic sinusoid of livers, and cortex, medulla, renal corpuscle (black arrows), proximal convoluted tubule (black boxes) of the kidney were clear, suggesting that 6-TG have no side-effects on mice under the way we treat it (**Figure 6G**). These results coherently indicated that 6-TG treatment could retard tumor growth *in vivo* through downregulation of the DNMT1 which could increase the DNA methylation of tumor promoting genes.

### Apoptosis Was Induced by CX-6258 HCl and MI-463 in MDA-MB-231 Cells

**Figure 7:**
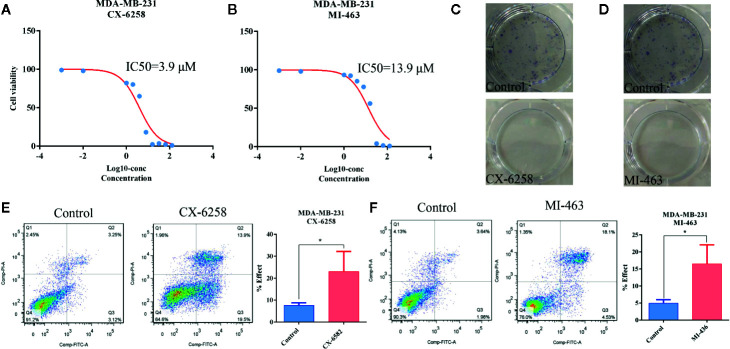
Apoptosis was induced by CX-6258 HCl and MI-463 in MDA-MB-231 cells. **(A, B)** IC50 values of CX-6258 HCl and MI-463 in MDA-MB-231 cells. X-axis presented the Log10-concentration, and the y-axis presented the cell survival rate. **(C, D)** Colony formation of MDA-MB-231 cells after treatment with CX-6258 or MI-463, and the same control group was used for the two groups. **(E, F)** Cell apoptosis was detected by flow cytometry. (All values from three independent experiments are quantified as mean ± SD, *p < 0.05).

Furthermore, CX-6258 HCl and MI-463, which are also used to treat leukemia, were selected stochastically to evaluate their effect on TNBC cells. As shown in **Figures 7A, B**, the IC50 of CX-6258 HCl and MI-463 showed low concentration of IC50 (3.9 and 13.99 μM, respectively) in MDA-MB-231 cells. We observed that, in comparison with the control (vehicle), CX-6258 HCl and MI-463 treatment led to significant inhibition of colony formation (**Figures 7C, D**). In contrast, flow cytometry assay showed that CX-6258 HCl and MI-463 treatment led to a marked increase of apoptotic cells (**Figures 7E–F**). Altogether, these data suggested that both CX-6258 HCl and MI-463 could efficiently inhibit cell proliferation and induce cell apoptosis in MDA-MB-231.

## Discussion

Over the past decade, advances in next-generation sequencing technology have greatly promoted the blowout growth of multi-omics data derived from various tumors. Researchers have attempted to investigate the pathogenesis of cancer using different tools including genomic DNA copy number arrays, DNA methylation, mRNA arrays, and microRNA sequencing (19, 20). Perou et al. synthetically analyzed the comprehensive molecular characterization of breast cancer and suggested that the PI3K and RAS–RAF–MEK pathways were amplified in this malignancy (21). Additionally, some controversial studies focused on the effect of viral infections on breast cancer development (22, 23). In our study, when we integrated the transcriptome map from leukemia and breast cancer cell lines, we found that besides the PI3K pathway, other pathways, including those involving proteoglycans in cancer, focal adhesion, and human papillomavirus infection were abnormally activated in TNBC. Many pathways, such as T-cell leukemia virus 1 infection, Herpes simplex infection, and Epstein–Barr virus infection, were elevated and enriched in TNBC than in other breast cancer types and lymphomas. Therefore, we speculated that TNBC was likely a cancer type with high expression levels of PI3K–AKT, proteoglycans in cancer, focal adhesion pathway, as well as a cancer type caused by virus (HTLV, HPV and EBV) infection similar to leukemia/lymphoma. Based on these findings, we proposed that leukemia drugs could have potential therapeutic effects on TNBC.

To verify our proposal, we chose 6-TG as a candidate drug to investigate its therapeutic role in TNBC. 6-TG is a classic DNMT1 inhibitor with remarkable success in clinical practice, especially in the treatment of ALL (17). Previous studies indicated that 6-TG could non-competitively inhibit human USP2 (24) thus plays a critical role in tumor cells survival. KY et al. reported that 6-TG-induced DNA mismatches can activate an MSH2/MLH1–BRCA1–ATR–Chk1 pathway, leading to a G2 arrest (25). Gu et al. found that MDA-MB-231 cells were sensitive to 6-TG (26), and low concentrations of 6-TG could inhibit cell growth. However, the underlying mechanism of 6-TG to suppress cancer cell growth and trigger apoptosis was unclear. It was well studied that *PTEN* acts as a negative regulator of PI3K signaling and subsequent AKT activation (27, 28). However, we did not find any research indicating that 6-TG has a function on tumor suppressor genes *PTEN* and *TSC1*. Interestingly, our results showed that 6-TG inhibited PI3K-AKT pathway *via* activating *PTEN*. Previous study showed Akt played a critical role in regulating Bad, Bcl-2, Cyto-c, Apaf-1, CASP9, and caspase-3 (18). In line with this, we found that inhibition of PI3K–AKT led to an increased expression of *BAD*, *TSC1*, and *CASP9* (**Figure 3C**). Consistently with other reports, we also observed that the expression of JUN was increased which might be caused by cellular stress (29, 30). Meanwhile, the finding that apoptosis-related genes including *TNF*, *DAXX*, *FADD*, *TNFRSF10B*, *CASP8*, *FADD*, *TRAIL*, and *TRADD* were all activated, indicated that 6-TG could induce cell apoptosis through extrinsic apoptosis pathways (activated by death receptors). Here our data indicated that 6-TG exhibited remarkable ability in inhibiting the highly activated oncogene pathways, inducing cell death and activating tumor suppressor genes in MDA-MB-231 cells. Furthermore, the effect of other two leukemia drugs on MDA-MB-231 cell growth was also tested. Consistently, both of them induced cell apoptosis and retarded cell growth in low concentrations, which suggested that our proposal might be reasonable. Although the underlying mechanisms of these two drugs inducing apoptosis need further investigation.

6-TG can facilitate the proteasome-mediated degradation of DNMT1 and reactivate epigenetically silenced genes in acute lymphoblastic leukemia cells (31). Therefore, we speculated that 6-TG might induce apoptosis by reducing DNA methylation in MDA-MB-231 cells. Maintenance of genomic methylation patterns is mediated primarily by DNMT1 (32). Su et al. showed that the epithelial–mesenchymal transition (EMT) could be inhibited by DNA methyltransferase inhibitors (5-azacytidine, decitabine, guadecitabine/SGI-110) in TNBC (33). Our previous findings also suggested that changes in the methylation levels could affect the EMT pathway in MDA-MB-231 cells (34). However, no analysis related to apoptosis and DNA methylation triggered by 6-TG in TNBC was reported. According to relevant research, *CASP8* and *CASP10* were both recruited to Fas by interacting with *FADD*, whereas *DAXX* binds directly to Fas, initiating a death pathway independent of *FADD*, which can enhance Fas-induced apoptosis by activating the JNK kinase cascade (35, 36). However, few studies reported the DNA methylation of these genes in TNBC. In fact, our results indicated the DNA methylation levels of tumor suppressor genes such as *PTEN*, *TSC1* and apoptosis-related genes such as *FADD*, *BAD*, *DAXX* were downregulated by 6-TG (**Figure 5C**). Importantly, when we focused on the pathway enrichment of downregulated genes, we found that they were enriched in TRAIL, FAS, and apoptosis pathways. This suggests that the mechanism underlying 6-TG activity in MDA-MB-231 was the regulation of DNA methylation in tumor suppressor genes and apoptosis-related genes. Based on these findings, we further found that the downregulation of DNA methylation of three CpG sites, *TSC1* (cg14350545), *FADD* (cg02794589), and *TRADD* (cg05178604) could significantly prolong the survival of breast cancer patients (DNA methylation survival curves in the TCGA). Therefore, the downregulation in DNA methylation of negative regulators of PI3K–AKT pathway *PTEN* explained why the abnormally activated pathways were inhibited. Meanwhile, 6-TG downregulated the DNA methylation levels of genes in the TRAIL and Fas pathways such as *DAXX* and *CASP8* through inhibiting the expression of DNMT1, subsequently inducing extrinsic apoptosis.

Altogether, our results demonstrate that 6-TG can markedly inhibit MDA-MB-231 cell growth, induce apoptosis through reactivating methylation-silenced genes in the apoptosis pathway and PI3K–AKT signaling pathways *via* blocking DNMT1 activity. Our data suggested a new strategy for detecting potential TNBC therapeutic drugs using bioinformatics analysis.

## Data Availability Statement

The datasets generated for this study can be found in the GEO number: GSE137418.

## Ethics Statement

The animal study was reviewed and approved by Jilin University Animal Care and Use Committee.

## Author Contributions

DZ: formal analysis, investigation, resources, data curation, writing (original draft, review, and editing), and visualization. XA: formal analysis, resources and writing (original draft and review),. QL: investigation, data curation. XM, MC, and HL: data checking, statistical analysis and investigation. NZ and XD: investigation, writing (review). HY: conceptualization, methodology, data analysis, writing (original draft and review). ZL: conceptualization, methodology, formal analysis, data curation, writing (original draft, review and editing), supervision, project administration, and funding acquisition. All authors contributed to the article and approved the submitted version.

## Funding 

This research was funded by the National Key R&D Program of China (No: 2017YFA0104400), Program for Changjiang Scholars and Innovative Research Team in University (PCSIRT, No. IRT_16R32) and Program for JLU Science and Technology Innovative Research Team (JLUSTIRT).

## Conflict of Interest

The authors declare that the research was conducted in the absence of any commercial or financial relationships that could be construed as a potential conflict of interest.
