# Supplementary material for: Thioguanine Induces Apoptosis in Triple-Negative Breast Cancer by Regulating PI3K–AKT Pathway
Source: Front Oncol. 2020 Oct 30;10:524922. doi: 10.3389/fonc.2020.524922 (PMC7662440; doi:10.3389/fonc.2020.524922)
Supplement: Supplementary file 1 [file DataSheet_1.pdf]

Supplementary Material

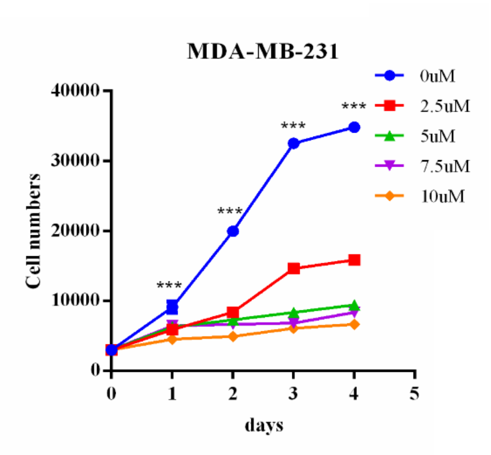

Figure S1. Cell proliferation curves of MDA-MB-231 cells.

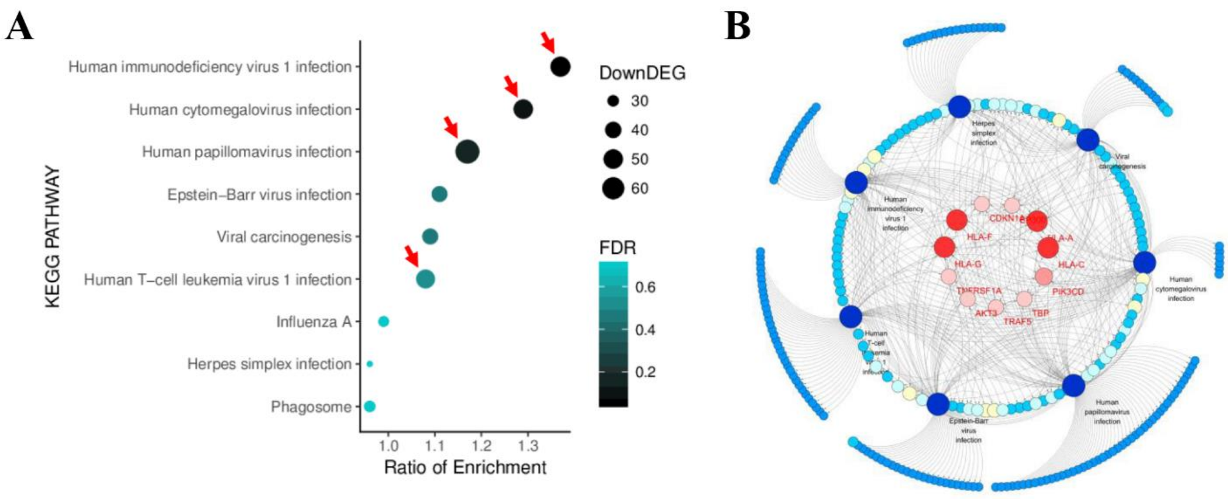

Figure S2. Virus infection pathway was inhibited by 6-TG. (A) The bubble plot of pathway enrichment of the downregulated genes. (B)The virus infection-related pathway gene network. The network was generated using 7 downregulated virus infection-related pathways and their enriched DEGs.

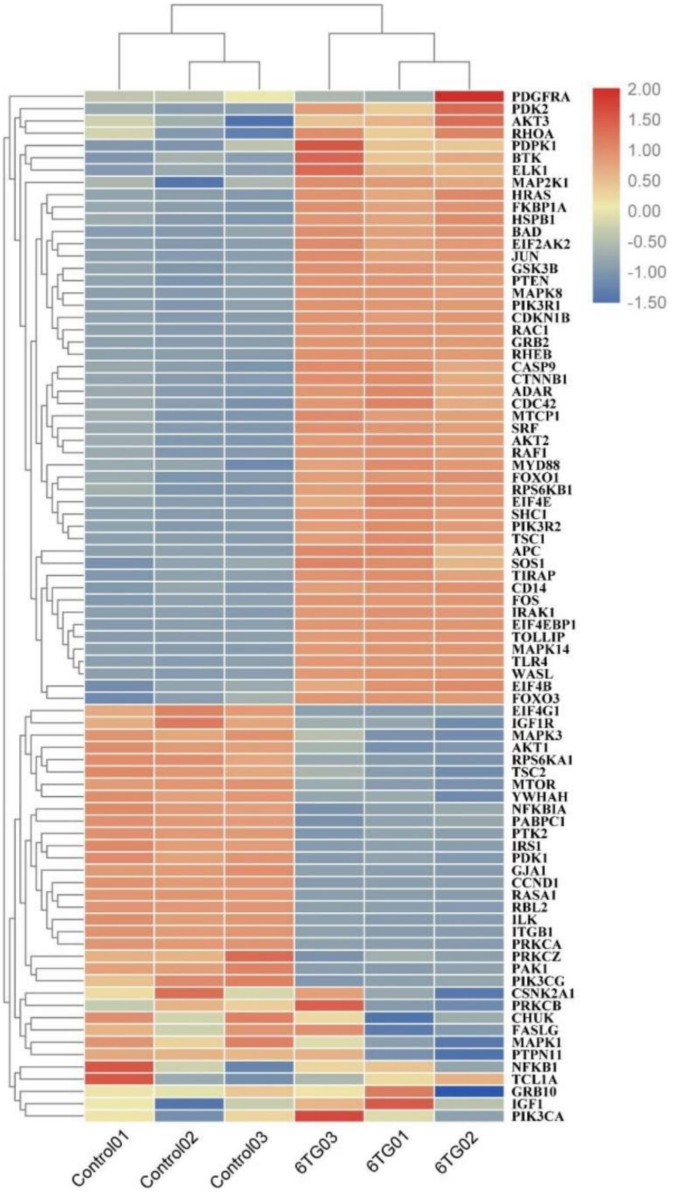

Figure S3. Heat map of PCR array analysis of gene expression concerning the PI3K-AKT pathway in MDA-MB-231 cells. The three groups on the left represent the control group, and the 6-TG group is on the right. Red and blue colors indicate higher expression and lower expression, respectively.

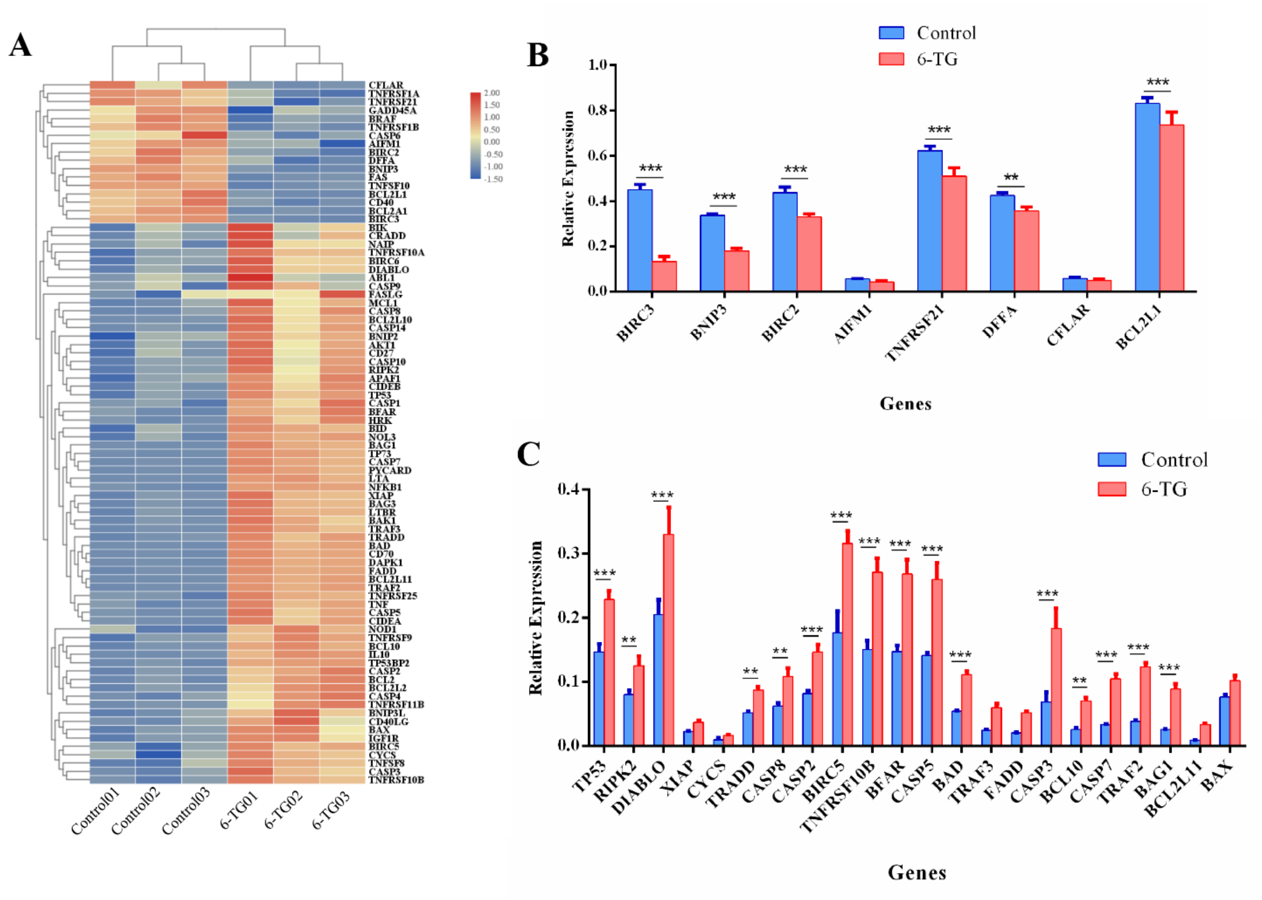

Figure S4. Apoptosis was induced by 6-TG in MDA-MB-231 cells. (A) Heat map of PCR array analysis of gene expression concerning the apoptosis pathway in MDA-MB-231 cells. (B) The relative expression of genes with significantly decreased from apoptosis array. (C) The relative expression of genes with significantly increased from apoptosis array.

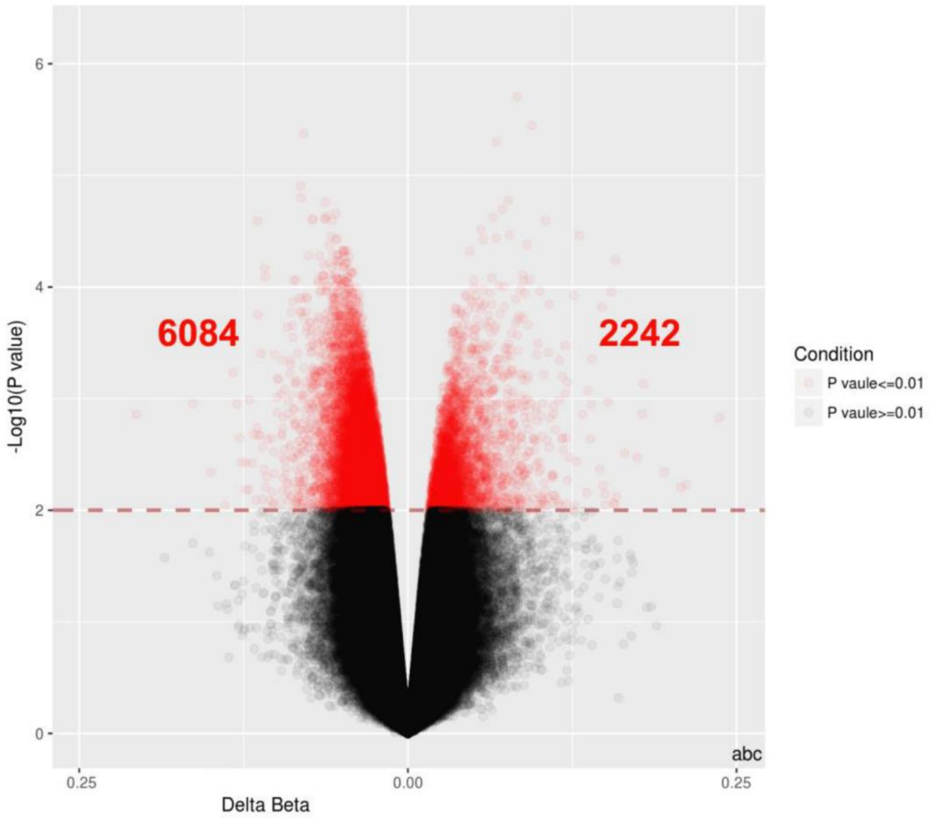

Figure S5. Volcano plot of differences in DNA methylation in MDA-MB-231 cells. Each point represents one gene. The x-axis represents the delta beta value (control group − 6-TG group), and the y-axis indicates −log10 of the P-value.

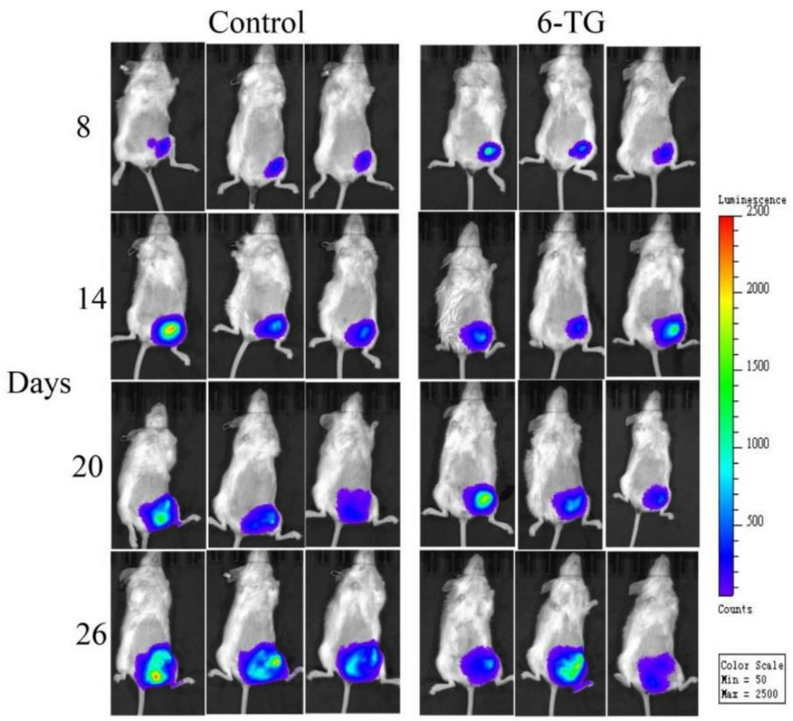

Figure S6. Tumor growth was detected by the bioluminescence imaging system. The left and right represent the control and 6-TG groups three mice, respectively, with three mice in each group. The labels on the left represent the days after implantation. The color scale represents the fluorescence intensity.
