# Supplementary material for: Thioguanine Induces Apoptosis in Triple-Negative Breast Cancer by Regulating PI3K–AKT Pathway
Source: Front Oncol. 2020 Oct 30;10:524922. doi: 10.3389/fonc.2020.524922 (PMC7662440; doi:10.3389/fonc.2020.524922)
Supplement: Supplementary file 2 [file Table_1.docx]

**Table S1:** **Primers sequences of PI3K-AKT signaling pathway PCR array.**

| **Gene** | **Forward primer (5’ to 3’)** | **Reverse primer (5’ to 3’)** |
| --- | --- | --- |
| AGT | CCAGCAGCAGATAACAAC | CACACTTAGACCAAGGAG |
| AKT1 | GGACAGAGGAGCAAGGTT | GGGACAGTCACCAAGAACT |
| ATF1 | GCTGCTGTCACTTCTATG | GACTTGCCAACTGTAAGG |
| BCL10 | GCCAGTCATTCAGCAGCAA | ACTCCATCAAGTGTTCCTCCAG |
| BCL2A1 | CCTACAGATACCACAACCT | TGTTCTGGCAGTGTCTAC |
| BCL2L1 | GTTGAGCCCATCCCTATT | CAGTCCTGTTCTCTTCCA |
| BCL3 | GGTCAACCCTTCTGGAAA | TAGGTGCATGTGCTTCTG |
| BIRC2 | AGGTCTGTCACTGGAAGAAC | GGCACATTCCTGGCATACTA |
| BIRC3 | CATCCGTCAAGTTCAAGC | GGTCTTCTCCAGGTTCAA |
| CARD11 | CTCAGCCGCTATATCAAC | GCACTTCATCTTCATCCT |
| CASP1 | GGTGCTGAACAAGGAAGA | CTGCCAGGTAACTGTCTT |
| CASP8 | CTGAGCCTGGACTACATTC | TTCAGGAAGGACAGATTGC |
| CCL2 | CCAGTCACCTGCTGTTAT | GCCACAATGGTCTTGAAG |
| CCL5 | GCCAACCCAGAGAAGAAA | GGACAAGAGCAAGCAGAA |
| CD27 | AGGTTGCTGCTATGAGAG | GCTGCTGTCTCTTCTTTG |
| CD40 | GGCTTCTTCTCCAATGTG | GGACCACAGACAACATCA |
| CFLAR | GTTACTTGGGAGGCTGAG | GGCTATTCGTAGGCACAA |
| CHUK | AAGCCTTACACAGCCACT | CCGAACTTCTCCTGACATCT |
| CSF1 | GCCTTCTAGTCACAGCCTCTAT | TGCTCTGATGCCACCAAGT |
| CSF2 | CCACTACAAGCAGCACTG | GGGATGACAAGCAGAAAGT |
| CSF3 | CTTCCTGGAGGTGTCGTA | TTCTGCTCTTCCCTGTCT |
| EGFR | CAAGGCACGAGTAACAAG | GGCAATGAGGACATAACC |
| EGR1 | TCTACTGGAGTGGAAGGT | GTGAAGAACTTGGACATGG |
| ELK1 | GGGAAGGAAGGGATTTGG | GGAGGGAGGGAACTGAAA |
| F2R | TTACGCTTCCTCTGAGTG | GGTTACTAGAGCAGGTATCC |
| FADD | CCTGCTTCTGAACTCAAG | CAGTGTCATAGTGAGGAAG |
| FASLG | TTACAGGCACCGAGAATG | CTTCCGTCATATTCCTCCA |
| FOS | GACTGATACACTCCAAGC | CTGCCAGGATGAACTCTA |
| HMOX1 | TTCAGCATCCTCAGTTCC | CCGTGTCAACAAGGATAC |
| ICAM1 | GCATTGTCCTCAGTCAGA | CTTCCTCTTGGCTTAGTCA |
| IFNA1 | GAATGCGGACTCCATCTT | TTCTGCTCTGACAACCTC |
| IFNG | GGAGACCATCAAGGAAGACAT | GACAGTTCAGCCATCACTTG |
| IKBKB | CTTGCTGAGTGACATTGC | ACCTCTGTTCTCCTTGCT |
| IKBKE | CAACGAGGAGCAGATTCA | TGTGACTAAGGACGCTTG |
| IKBKG | GGACTCCTCTAGTTCAGA | GTGCCTATTCATCCAACAG |
| IL10 | TTCCATTCCAAGCCTGAC | CTCCCTGGTTTCTCTTCC |
| IL1A | ACCAACCTCCTCTTCTTCTG | AGCACACCCAGTAGTCTTG |
| IL1B | GGATATGGAGCAACAAGTGG | CGCAGGACAGGTACAGAT |
| IL1R1 | GAGACTGACACCTCACTGA | CTTCGGACACTAACTTCTGG |
| CXCL8 | AATCTGGCAACCCTAGTCTG | GTGAGGTAAGATGGTGGCTAA |
| IRAK1 | CAGACAGGGAAGGGAAACAT | TTGGACACGCAAGAGGAC |
| IRAK2 | CCAGCAGATTCCATTACC | AGCAAATACAACCCAGAG |
| IRF1 | AGATGAGGATGAGGAAGG | CCAGGTTCATTGAGTAGG |
| JUN | GTGCCAACTCATGCTAAC | GTTCTCAAGTCTGTCTCTCT |
| LTA | TATCCACCCACACAGATG | AGAATGGAGGCAGAATGG |
| LTBR | GCACCTATGTCTCAGCTA | GCAGATGGTCAGGTAGTT |
| MALT1 | CCGATCACCAGGCATAAC | GTCAGGAAGGCAGTTGTG |
| MAP3K1 | GGTCAACAGTATGGAAGGA | ACAACGAAGAGCCACATC |
| MYD88 | TTCTCTCCCTCTCTCCTT | CACAGACACCTAAGACCAT |
| NFKB1 | CCACCTTCATTCTCAACTTG | AATCCTCCACCACATCTTC |
| NFKB2 | GAATGGACAAGACAGCAG | CTCATCATCCTCATAGAACC |
| NFKBIA | ATCCTGAAGGCTACCAAC | CTCCTGAGCATTGACATC |
| NFKBIB | GAGAAGGAAGAAGAGGAG | ACCATCTCCACATCTTTG |
| NFKBIE | CCAACCAGTAGGAAGCAT | TTTCAGGGTCCTCAACAG |
| NOD1 | GCCGCTTGCTACTTTAAC | GGATGTGAGTGACCAGAA |
| PSIP1 | CAGCAATGAGGATGTGAC | GGAATCTTGACTTCTGTAGC |
| RAF1 | GTGGACTGGAGTAACATCA | GGCGTGAGGTGTAGAATA |
| REL | TTGACGACTGCTCTTCCT | CATCTCCTCCTCTGACACTT |
| RELA | CCTCTTCTCAAGTGCCTTA | AGAGCAAGAGTCCAAGTG |
| RELB | GGAAGAAGGAGATTGAGG | CACCACATTCATGTCTAC |
| RHOA | TTCAGCAAGGACCAGTTC | CCCACAAAGCCAACTCTA |
| RIPK1 | CAAGACGAAGCCAACTAC | TCTCCTTTCCTCCTCTCT |
| STAT1 | TCAGACCACAGACAACCT | GTCGCCAGAGAAGATGAA |
| TBK1 | AGCCATGACCGACCAATATG | AGCCACCGTTCTACCATT |
| TICAM1 | GTAGAAGATACCACCTCTC | TAGATGAAGGAGGAGGAG |
| TICAM2 | ATTCCTGCCCTCTTTCTC | CCTCTGTTGTATTGCTGTG |
| TIMP1 | CTTCACCAAGACCTACACT | CGTCCACAAGCAATGAGT |
| TLR1 | GCACTTGGACCTGTCATT | CTCCTAAGACCAGCAAGAC |
| TLR2 | GCTGCCATTCTCATTCTTC | GGTAGGTCTTGGTGTTCAT |
| TLR3 | TTCACCATTCCAGCCTCTTC | GCCGTGCTAAGTTGTTATGC |
| TLR4 | TGCCTTCACTACAGAGACT | GGGACACCACAACAATCA |
| TLR6 | CCAGTCCGTATTATCCATCTC | CCTTCTACCATGCTGAGTC |
| TLR9 | GGAGCTACTAGGCTGGTA | AAGGATGCTTCACACTCG |
| TNF | CATCTGGAATCTGGAGAC | CTGGAAACATCTGGAGAG |
| TNFAIP3 | CTGGCAACTGGAGTCTCT | ATGGGTGTGTCTGTGGAA |
| TNFRSF10A | GGGATGGTCAAGGTCAAG | CAACAGCAACGGAACAAC |
| TNFRSF10B | GGCTCACTTCTGGTTATC | CCATTCTAGGTCCTGTTG |
| TNFRSF1A | TGTTAAGGGCACTGAGGA | CCGTTGGTAGCGATACAT |
| TNFSF10 | CAGAGGAAGAAGCAACAC | GGATGACCAGTTCACCAT |
| TNFSF14 | TTCAGAGTCAGGAAGGATG | GCTAAGGGAGAGTCAATCA |
| TRADD | CCAGCCCTTACAGTTTCA | GCAGGCAAGATTGATTCC |
| TRAF2 | GATTGAAGCCCTGAGTAG | CTCCATCTCCAAGACCTT |
| TRAF3 | GATTCGCAGCCTCTTCTC | GCTACTACAGACGCAACTT |
| TRAF6 | GCCACCTACAAGAGAACA | CGGACCTCTGAGATATACC |
| CCL20 | GAATCAGAAGCAGCAAGC | TGTCACAGCCTTCATTGG |
| CD83 | TCCTGTAGCCTTCTGTAG | CCGTGTGTCCATATCTTG |
| CXCL2 | CGAAGTCATAGCCACACT | TCCTTCCTTCTGGTCAGT |
| CXCL3 | GTCATAGCCACACTCAAG | CTTCTCTCCTGTCAGTTG |
| STX11 | CCTTCCTCTGTGTCAACTCTTAGC | GTCATAAGCAAGTCAGGCAGTGA |
| TIFA | AAGGAAGATGGCGAGTCA | AGGAGCAGAGCGAATAAGT |
| TNFAIP2 | GCTTGACCTCTCACTGTT | GAACTGGAAGACCTCGTT |
