# Supplementary material for: Thioguanine Induces Apoptosis in Triple-Negative Breast Cancer by Regulating PI3K–AKT Pathway
Source: Front Oncol. 2020 Oct 30;10:524922. doi: 10.3389/fonc.2020.524922 (PMC7662440; doi:10.3389/fonc.2020.524922)
Supplement: Supplementary file 3 [file Table_2.docx]

**Table S2:** **Primers sequences of apoptosis PCR array.**

| **Gene** | **Forward primer (5’ to 3’)** | **Reverse primer (5’ to 3’)** |
| --- | --- | --- |
| ABL1 | GTGAGAGTGAGAGCAGTC | CCATCAGAAGCAGTGTTG |
| AIFM1 | TCTGTCTGCCATTGATAGG | CAGTTCGCTACCAAGGAA |
| AKT1 | GTGGCTATTGTGAAGGAG | CATTCTTGAGGAGGAAGTAG |
| APAF1 | CCAGAGGCTTCCACTTAA | CCCAAGAGTCCCAAACAT |
| BAD | GAATATGGGCGGAAGTAC | GAAGGGAATCTGGGTCAG |
| BAG1 | GCAAGGTTGTAGAGTAAGC | GTTGGTTGAGAGGCAGTA |
| BAG3 | GGTGTCAGGAAGGTTCAGA | TGGGCTGGAGTTCATAGAC |
| BAK1 | TCTGGCACAGTGTAATCC | TAGACAGGTGAGGAGCAT |
| BAX | CTTCTGGAGCAGGTCACA | CAGCAGGGTAGATGAATCG |
| BCL10 | GCCAGTCATTCAGCAGCAA | CTCCATCAAGTGTTCCTCCAGTA |
| BCL2 | TGATGCCTCTGCGAAGAACCT | GCCATGCTGATGTCTCTGGAATC |
| BCL2A1 | TGTTGCGTTCTCAGTCCAA | CCAGTTAATGATGCCGTCTTC |
| BCL2L1 | GTTGAGCCCATCCCTATT | CAGTCCTGTTCTCTTCCA |
| BCL2L10 | TCAGGTGTGAGGACAAGAA | AGGTTCGCACTCTTATCCA |
| BCL2L11 | AAGAGTTGCGGCGTATTGGAGA | TGGTCTTCGGCTGCTTGGTAAT |
| BCL2L2 | TAGGCGATTGGAAGAGTG | CCTGGAACGACTACATCT |
| BFAR | GAACACAATGGACCTTGAGAG | AACCAGGATGTCGTAGCA |
| BID | CCTTGCTCCGTGATGTCTT | CCGTTCAGTCCATCCCATT |
| BIK | GACATCTTGATGGAGACC | CCTCTTCAGAGTCAGTCA |
| BIRC2 | GGTCGCAATGATGATGTC | GAACTCACACCTTGGAAAC |
| BIRC3 | ATGGTGGACTCAGGTGTT | GCTTGAACTTGACGGATGA |
| BIRC5 | AACAGTGGCTGCTTCTCT | CCTCACTTCTCACCTGGTAA |
| BIRC6 | GGAACCTACTGATGAACCT | GCAAGAGTGACTGACAATG |
| BNIP2 | CTAGTGATGGCTCTGTATTGTC | TTCCTCTGCTGCTGTGTATT |
| BNIP3 | ATCTCTGCTGCTCTCTCA | TCAGACTCCAGTTCTTCATC |
| BNIP3L | GCAATGATAATGGCAATGG | CCTCTGGAACTACTCTGT |
| BRAF | CTATATGCTTGTGCTTCTCC | TTGCTACTCTCCTGAACTC |
| CASP1 | ACATCCTCAGGCTCAGAAG | CGGCTTGACTTGTCCATT |
| CASP10 | AAGCCGAGTCGTATCAAG | CTCTGTGGTTCCGATTCA |
| CASP14 | CGTTCCCTGGTATCCTCAA | TGGTGTCTGGTGGCATAT |
| CASP2 | TCTGGAGAAGGACATCATC | CAGCAAGTTGAGGAGTTC |
| CASP3 | GTTCATCCAGTCGCTTTGT | TTCTGTTGCCACCTTTCG |
| CASP4 | CCATAGAACGACTGTCCAT | GGTGCTCCTTGAAGTTGA |
| CASP5 | CGGATGTGCTGCTTTATGAC | GCCTGGACAATGATGACCTT |
| CASP6 | GGCTTGTTCAAAGGAGAC | CCTCAGTTATGTTGGTGTC |
| CASP7 | ACTGACTGAGATGGAGTGAG | AAGGTGCTGGTGGAAGAT |
| CASP8 | TCATAGAGATGGAGAAGAGG | AGAGTCCGAGATTGTCAT |
| CASP9 | AGTGACAGACAGGCTCTT | GCAATCCACGGCATTCAT |
| CD27 | AGGTTGCTGCTATGAGAG | GCTGCTGTCTCTTCTTTG |
| CD40 | TCGGCTTCTTCTCCAATG | GGACCACAGACAACATCA |
| CD40LG | CGTCCTTTGGCTTACTCA | CTTAACCGCTGTGCTGTA |
| CD70 | TGACCACTGCTGCTGATTAGG | TCTTGTCCTGCCACCACTA |
| CFLAR | GTTACTTGGGAGGCTGAG | GGCTATTCGTAGGCACAA |
| CIDEA | GAATAGCGAGAGTCACCTT | GTAGGACACGGAGTACAT |
| CIDEB | GCCTCCATTCCTACTAAG | ATCTAGCCTGTACTGTCT |
| CRADD | ACCTCTACCAGGAAGGAATCT | ATCCAGCAGGAGCATTGTT |
| CYCS | AAGTCTCTTCTAGGCACTGT | GGATATGAACACCAGGACAATG |
| DAPK1 | GAGGCAGATATGTGGAGTAT | CGTAGTTGACAGCGGATA |
| DFFA | CTGTGGCGTTGTGTTATTG | TCTCTGGAAGGTGCTCTAA |
| DIABLO | TGTGACGATTGGCTTTGG | ACACTGCTCTCCTCATCAA |
| FADD | TCTGAGACTGCTAAGTAGGG | AGAGAGTGCTGTGTGTCA |
| FAS | ACTCACCAGCAACACCAA | CTTCCTTTCTCTTCACCCAAAC |
| FASLG | GTTACAGGCACCGAGAAT | ACATTAGGTGAGTTGAGGAG |
| GADD45A | GGTGACGAATCCACATTCATCTC | CCATTGATCCATGTAGCGACTT |
| HRK | GCTGGTGGAATAAGAGGTA | GCTAGGACGAGTCAAGAA |
| IGF1R | CAGTGAGGTTGAGGTGAGAG | CCCGCCTTCTGGTTTGAT |
| IL10 | CTTCCATTCCAAGCCTGAC | CCAAGCCCAGAGACAAGATA |
| LTA | GCCTTCGCTCTGTAGAACTTGGA | TGGTCAGAATGGAGGCAGAATGG |
| LTBR | CTAAATGTAGCCGCATCC | GCAGATGGTCAGGTAGTT |
| MCL1 | GAAGGTGGCATCAGGAAT | GTGGTGGTGGTTGGTTAA |
| NAIP | ACAGGAACTGCTTCTCAC | TCAGGCACAGGAACTTATC |
| NFKB1 | CTGTTGTCATTGCTGTTGTC | ATGCTGTGGTCAGAAGGA |
| NOD1 | CCTCACCTCATTCCAACA | GCCACATCCTCAACTCTT |
| NOL3 | GGGAAGTGAGACTAGAAGAGG | CCAGAGAGAAGCCAGACAAT |
| PYCARD | CTTTATAGACCAGCACCG | GTACTGCTCATCCGTCAG |
| RIPK2 | CCTTAACCAGTCGCTAGATG | TCCTTGGATGTCAGTAGTGT |
| TNF | CTCAACCTCTTCTGGCTCAA | CGAAGTGGTGGTCTTGTTG |
| TNFRSF10A | TACGCCCTGGAGTGACAT | CCAACAGCAACGGAACAAC |
| TNFRSF10B | GGAGGAGACATACAATGG | CCTGTAGAAGTTGCCAAT |
| TNFRSF11B | CCCAGAGCGAAATACAGTTTG | CTGAGTTAGCAGGAGACCAA |
| TNFRSF1A | TTAAGGGCACTGAGGACT | CCGTTGGTAGCGATACAT |
| TNFRSF1B | CGGCTCAGAGAATACTATG | GAGGTCTTGGTACAGAAG |
| TNFRSF21 | GTGCCTTCTAGTGTGATGA | GTTGTCTGTCTCCTTGGT |
| TNFRSF25 | CCTTCTACTGCCAACCAT | CCACAGTCAGTATCTCTG |
| TNFRSF9 | GCTCTTCCTGCTGTTCTT | AGTTCACATCCTCCTTCTTC |
| TNFSF10 | CAGAGGAAGAAGCAACAC | GATGACCAGTTCACCATTC |
| TNFSF8 | TCAGACAGAGACAGCAAGACA | CTTCCTTCAGACAGACCATTCAG |
| TP53 | TCAGCATCTTATCCGAGTG | TACAGTCAGAGCCAACCT |
| TP53BP2 | GCGTCCGTTCTCAATGTT | CTTCACTGCTCTGGTTCTTC |
| TP73 | GCTTCATCCTAGAGACTGT | GCTGCTCACTACTATCCA |
| TRADD | CCAGCCCTTACAGTTTCA | GCAGGCAAGATTGATTCC |
| TRAF2 | CAGGTACGGCTACAAGAT | CACAAAGAAGAGGGACAG |
| TRAF3 | GAGCCTGCGTTTCATTTC | AGCGATCCTGGACTTCTA |
| XIAP | GCTGGACTCTACTACACAGGTAT | CAGGCACGATCACAAGGTT |
