# Supplementary figures and images for: Thioguanine Induces Apoptosis in Triple-Negative Breast Cancer by Regulating PI3K–AKT Pathway
Source: Front Oncol. 2020 Oct 30;10:524922. doi: 10.3389/fonc.2020.524922 (PMC7662440; doi:10.3389/fonc.2020.524922)

**Table S3: Antibodies of antibody array.**


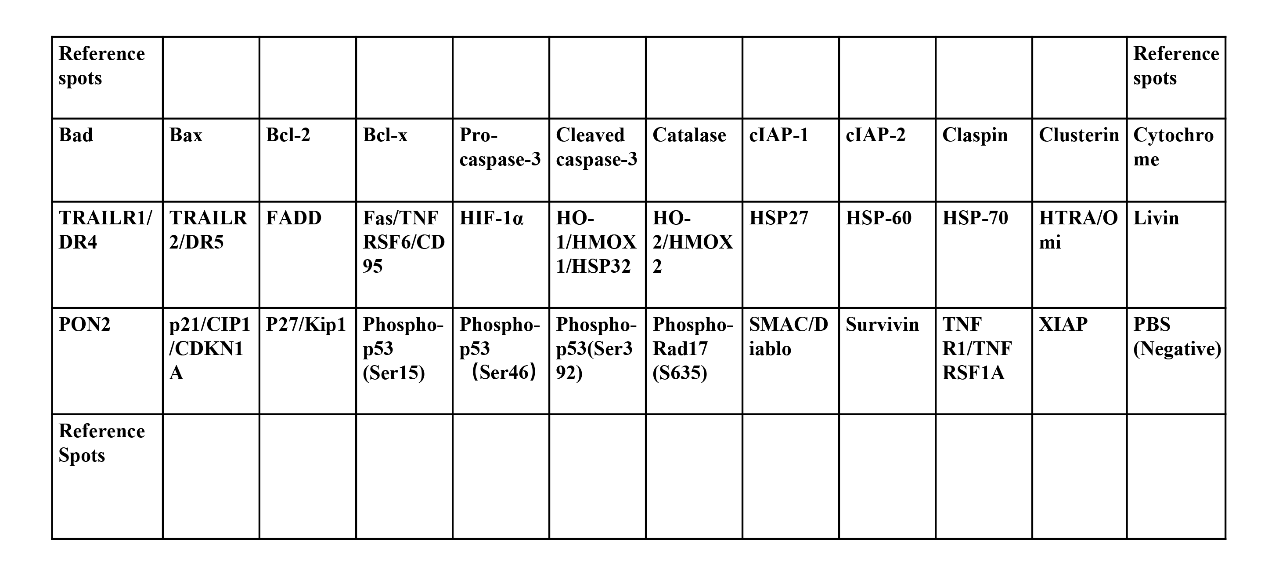

Supplement: Supplementary file 4 [file Table_3.docx]
